# Supplementary material for: The UCP2/PINK1/LC3b-mediated mitophagy is involved in the protection of NRG1 against myocardial ischemia/reperfusion injury
Source: Redox Biol. 2025 Jan 23;80:103511. doi: 10.1016/j.redox.2025.103511 (PMC11808529; doi:10.1016/j.redox.2025.103511)

Supplement Figure 1

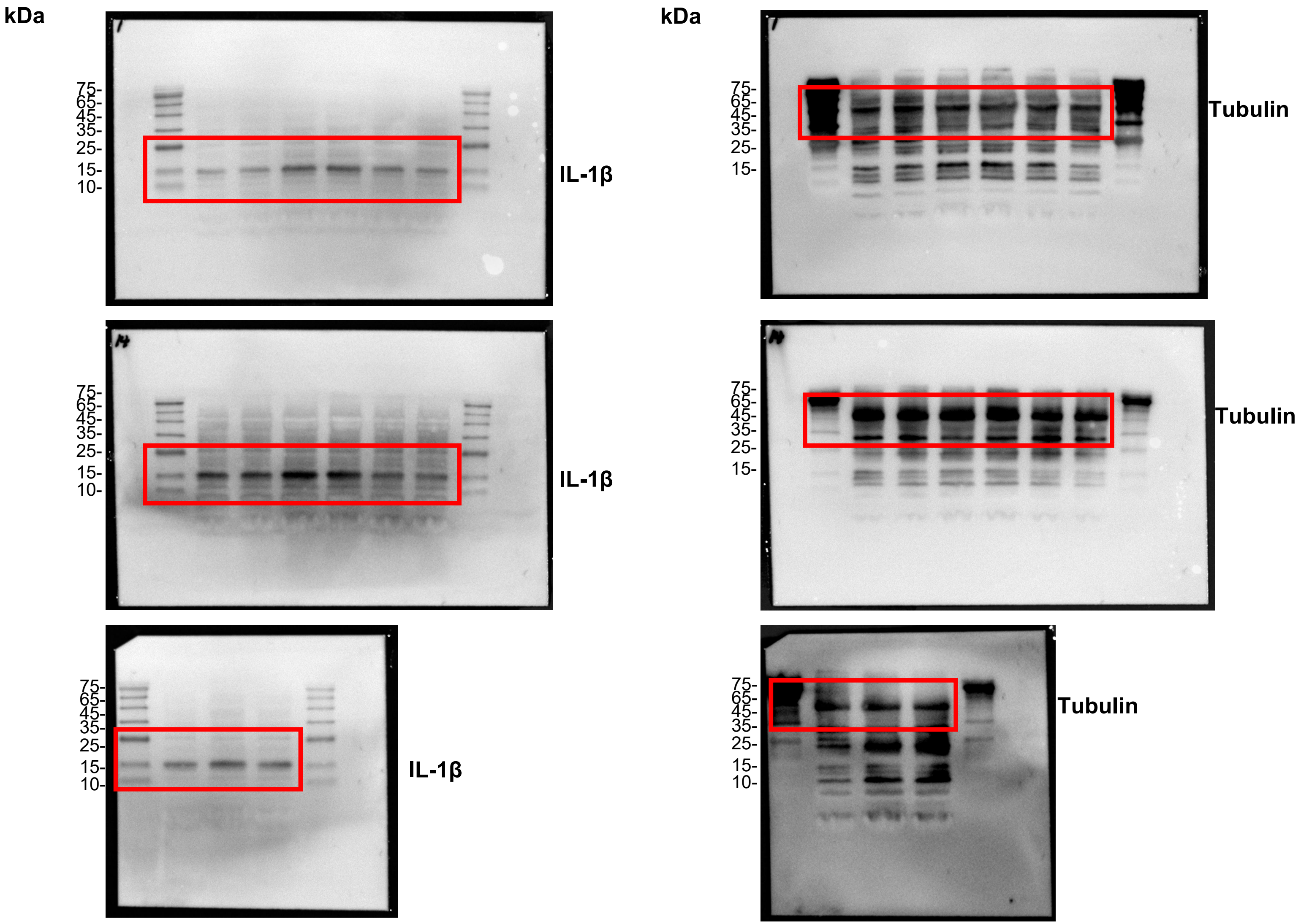

Supplement Figure 2

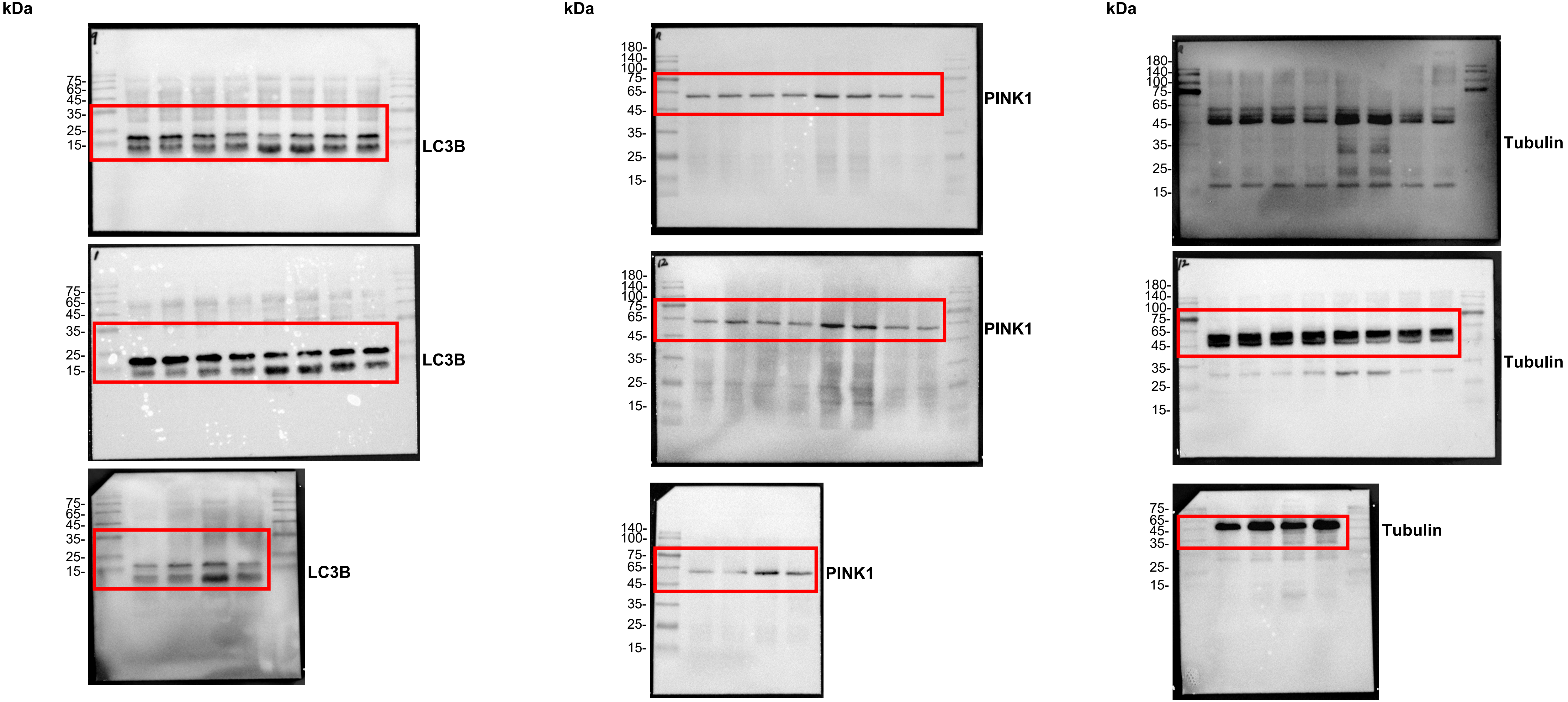

Supplement Figure 3

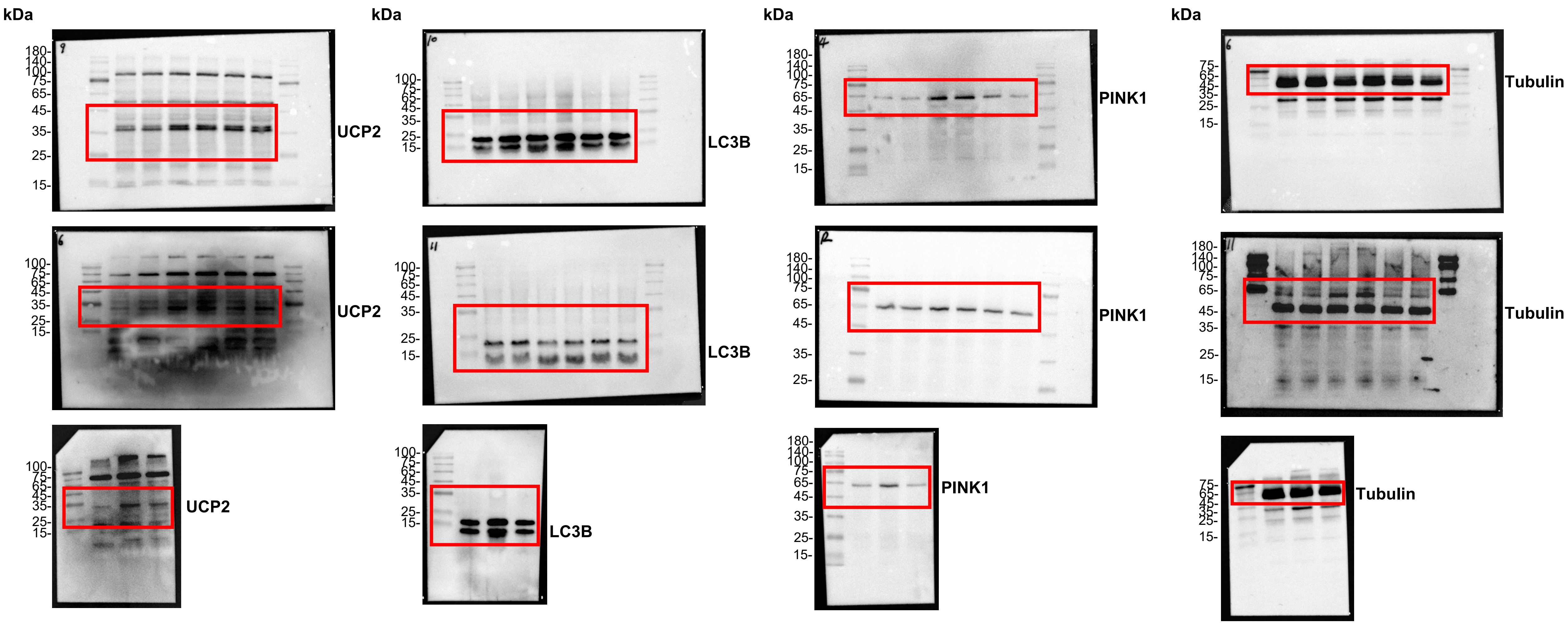

Supplement Figure 4

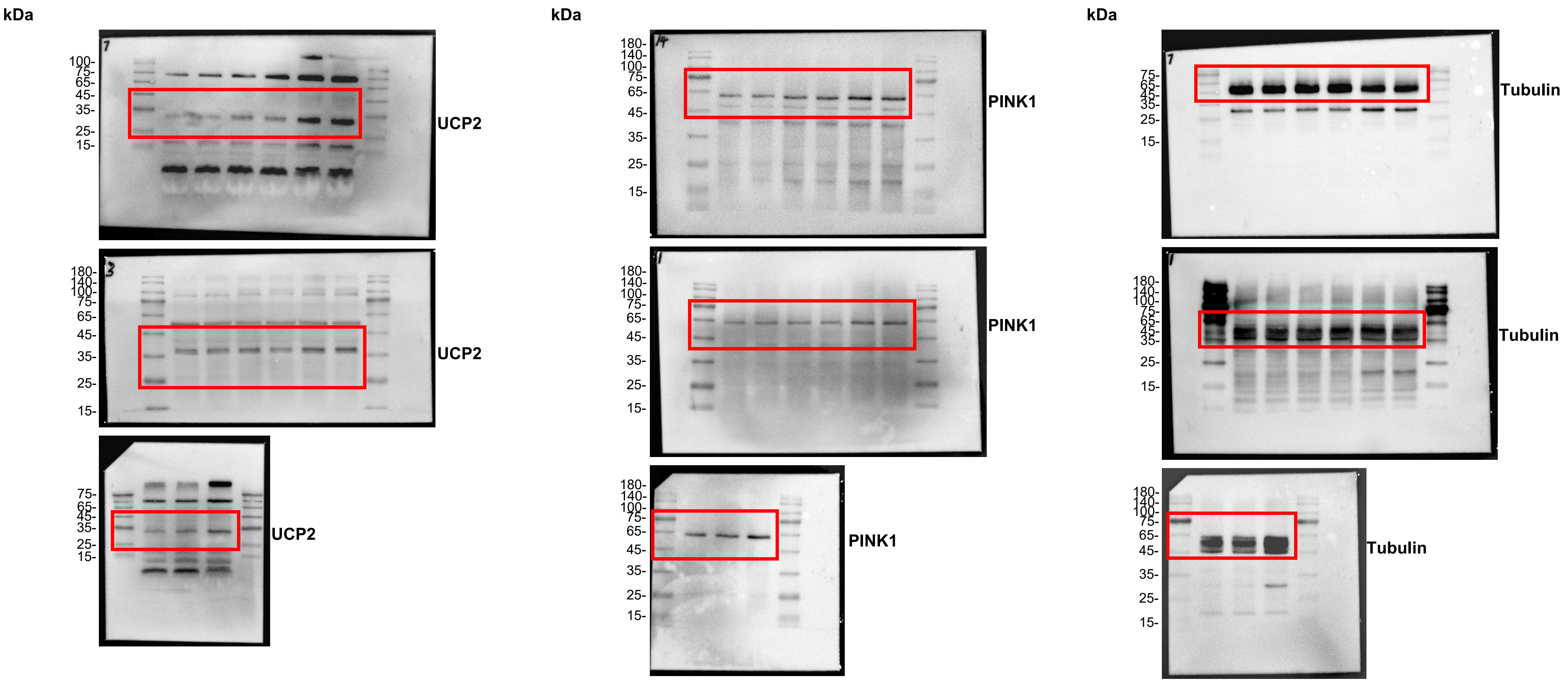

Supplement Figure 5

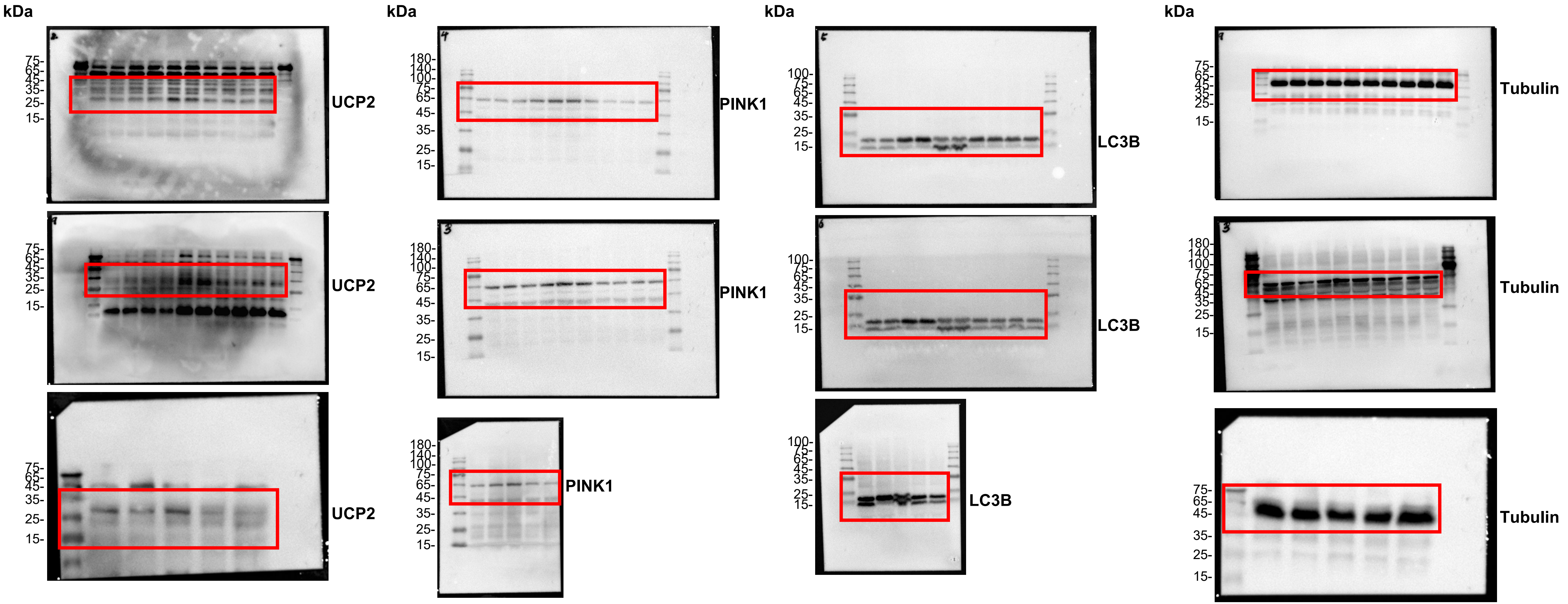

Supplement Figure 6

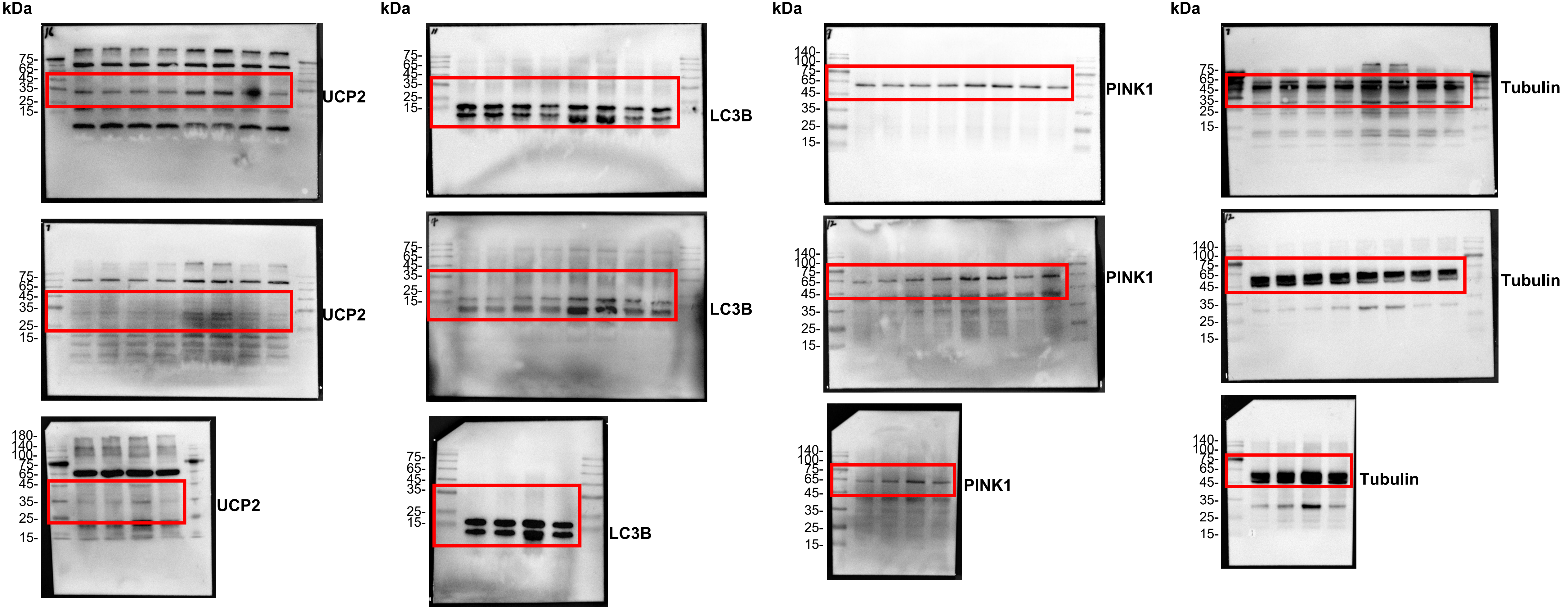

Supplement: Multimedia component 1 [file mmc1.pdf]
